# Epidemiologic Properties of Pediatric Fractures in a Metropolitan Area of Turkey

**DOI:** 10.1097/MD.0000000000001877

**Published:** 2015-10-30

**Authors:** Ahmet Issin, Nizamettin Kockara, Ali Oner, Vedat Sahin

**Affiliations:** From the Mengucek Gazi Education and Research Hospital Orthopedics and Traumatology Department, Erzincan University, Erzincan (AI, NK); Orthopedics and Traumatology Department, Metin Sabanci Bone and Joint Diseases Education and Research Hospital, Istanbul (AO); and Mengucek Gazi Education and Research Hospital Orthopedics and Traumatology Department, Erzincan University, Erzincan, Turkey (VS).

## Abstract

Occurrence of fractures is highly dependent on lifestyle. Domestic data should be used when needed. In this cross-sectional study, the authors aim to find the most recent distribution of pediatric fracture types and the attributes of fracture occurrence within a large sample size in a metropolitan area of Turkey.

This study consists of 4879 pediatric age patients with a fracture who took advantage of the emergency service of a trauma center in a metropolitan area between March 2010 and December 2013 (1397 days). Date, hour, age, sex, fracture type, and social security status of the patients were studied.

A total of 65% of the patients were men and 35% were women. A total of 81% of the fractures were in the upper extremities, whereas 19% of them were in the lower extremities. In 22 patients (0.5%), there were open fractures. Fractures showed some seasonal, daily, and circadian variations. Different types of fractures showed some specific patterns in different age groups. Ankle, elbow, and shoulder fractures were more common in girls, whereas wrist and forearm fractures were more in boys and the difference was statistically significant (*P* < 0.05).

Fractures in pediatric ages vary depending on the age, sex, season, and the hour of the day. Types of fractures show some obvious patterns especially depending on the age. This data can be useful in making optimizations in fracture care units. Considering these specific patterns would enable more effective planning of providing preventive measures for pediatric injuries.

## INTRODUCTION

Fractures occur during daily activities and they strongly relate to the lifestyle of the people in a specific region. Lifestyles may change drastically between different countries and even in time within the same country. For this reason, determining up to date, domestic data is important.^1–5^ Risk assessment and incidence studies are mandatory to watch the trends during time. For these types of population-based studies some requirements, however, should be met. Current studies in the literature, used databases of sole hospitals, which have the only responsible fracture care units in defined and populated areas, where the population represents the whole country.^6–8^ Alternatively, they used collaborative general medical registry databases that have significant number of patient records that also represent the general demographics.^3^ With the current setup, these type of studies cannot be done in Turkey. The most convenient way to find the generalizable results of the properties and the distribution of pediatric fractures in Turkey is using a large sample size acquired in a relatively shorter period inside the most generalizable population. More than 77% of the population of Turkey is living in metropolitan cities. Thus, large data from a metropolitan area would yield more reliable results. There are very few studies, with lower sample sizes, about the epidemiology of pediatric fractures in Turkey.^9,10^ This study aims to satisfy the curiosity about the domestic epidemiological properties of pediatric fractures in Turkey.

### Patients and Methods

In this study, we used the computer database of a trauma center to find out the pediatric patients with a diagnosis of fracture. Registry search was limited to the dates between March 2010 and December 2011. All data were acquired by the BilMedical HBYS (Bilmed, Istanbul, Turkey) software from the same database. All relevant fracture codes in the International Classification of Diseases, 10th revision list starting with S42, S52, S62, S72, S82, and S92 were used to find the patients with a diagnosis of a fracture. Subcodes with suffix 9, which indicates an unspecified location, were excluded. The patients with the same fracture codes were saved in separate excel files and the cases were sorted by name to ease the duplicate search and diagnosis crosscheck. Duplicated registries because of subsequent applications of the same patients because of fracture complications and neurovascular check routines were excluded. Patients were checked whether they had different diagnosis codes on subsequent admissions and if so diagnoses were corrected according to the doctors’ additional notes—if they existed—or the most repeated code on the follow-up examinations when necessary. Fracture types were tagged according to the anatomic site and grouped under more generic titles. Age, sex, social security status, type of the fracture, date, and the hour of assessment were extracted and studied. Ethical approval was not necessary for this study because the study was noninterventional but retrospective and observational.

### Statistics

Patient records were acquired from BilMedical HBYS (Bilmed, Istanbul, Turkey) hospital database as an Excel document. These data were imported to IBM SPSS Statistics 22 (SPSS Inc., IBM, Chicago, IL) software. Distributions of the fracture types were found. Line graphs and histograms were drawn to visualize the patterns. Z test was used to compare the proportions between 2 independent samples. *T* test was used to compare the means of 2 independent samples.

## RESULTS

### Distribution of the Patients

A total of 212.383 patient records were found between March 2010 and December 2013. A total of 21.563 (10.2%) patients were below the age of 16. Among these pediatric patients, 4879 had diagnosed with a fracture (22.6%).

### Health Insurance

Of the patients, 96.5% had valid health insurance and 1.4% were green card “Yeşil Kart” holder.

### Sex and Age

A total of 3167 (≈65%) were boys and 1712 were (≈%35) girls. Boy to girl ratio was 1.84. The mean age for the boys was 7.1 and for the girls it was 8.6, which is statistically significant (*P* = 0.001).

**FIGURE 1:**
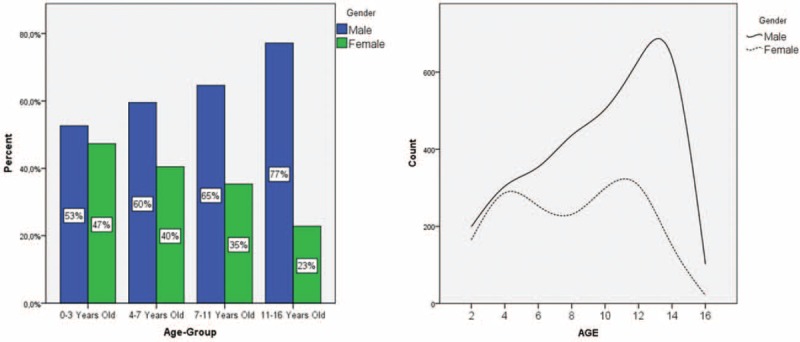
Sex–age distribution of the patients.

Boy to girl ratio was 1.1 between the ages of 0 and 3. It increases gradually and between the ages of 11 and 16 boy to girl ratio was 3.4 (Fig. 1).

### Season

**FIGURE 2:**
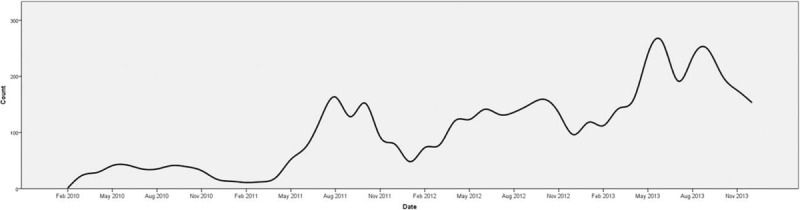
Distribution of the patients during the period.

There was an obvious seasonal variation (Fig. 2).

**FIGURE 3:**
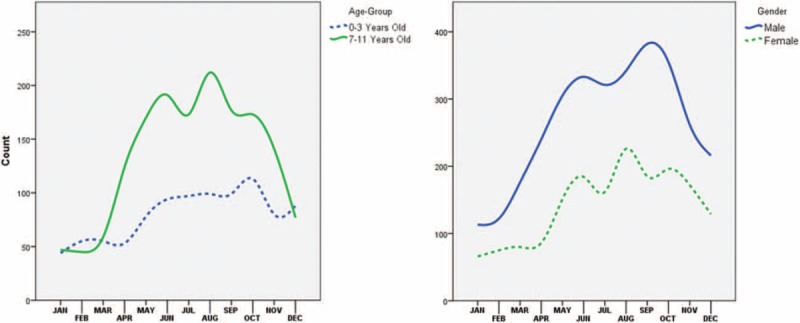
Monthly distribution of the patients depending on the age and sex.

A total of 31.8% of the fractures occured in the autumn, 32.1% in the summer, 21.3% in the spring, and 14.8% in the wintertime. This pattern did not change within the boys or the girls but the patients below the age of 3 did not show such a strong correlation (Fig. 3).

### Day of the Week

There were more fractures at the beginning and at the end of the week. On Mondays, it was 15.2% and on Fridays and Saturdays, it was 14.9%. Sundays had the lowest ratio of 13.2%.

### Hour of the Day

**FIGURE 4:**
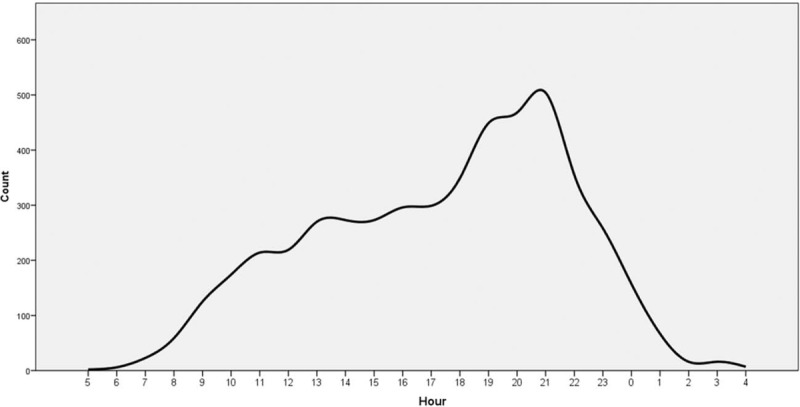
Patient admissions depending on the hour of the day.

Admission of children suffering from fractures to the emergency service was lowest between 02:00 and 07:00. It gradually increased after 07:00 and reached a plateau between 12:00 and 17:00. There was another increase after 17:00 and reached its peak at 21:00 and then gradually decreased. Admission rates at 11:00 and 23:00 were similar (Fig. 4).

### Fractures by Age Group

**FIGURE 5:**
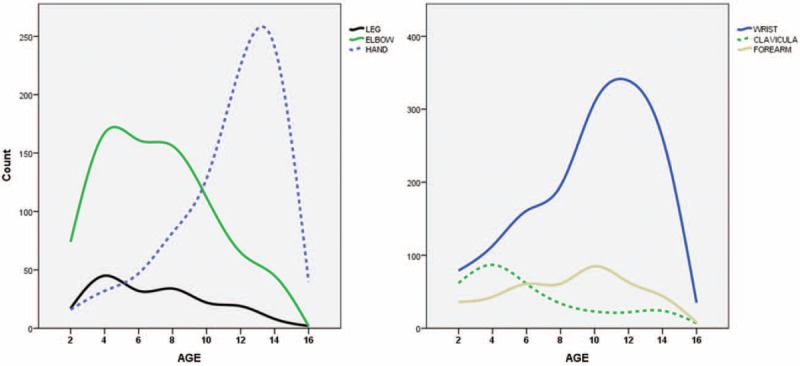
Relations between fracture types and the age.

It is clearly visualized that certain age groups have certain fracture types (Fig. 5). Hand and wrist fractures were seen at older ages, whereas clavicle and elbow fractures showed an early peak. Fractures of the leg were seen more in the early ages, but it showed an irregular distribution for all ages.

### Fracture Location

A total of 19% of the fractures were in the lower extremities, whereas 81% of them were in the upper extremities. This distribution did not change in both sexes (*P* = 0.66). Distal radius fracture (26.4%) was the most common fracture followed by distal humeral fractures (13%), finger fractures (11.5%), forearm both bone fractures (8.6%), clavicle fractures (6.6%), and metatarsal fractures (4.3%) (Table 1). Fracture distributions within sexes were calculated and significant differences were checked using z test (Table 1). Ankle, elbow, and shoulder fractures were more common in girls, whereas wrist and forearm fractures were more in boys and the difference was statistically significant (*P* < 0.05) (Table 2).

**TABLE 1:**
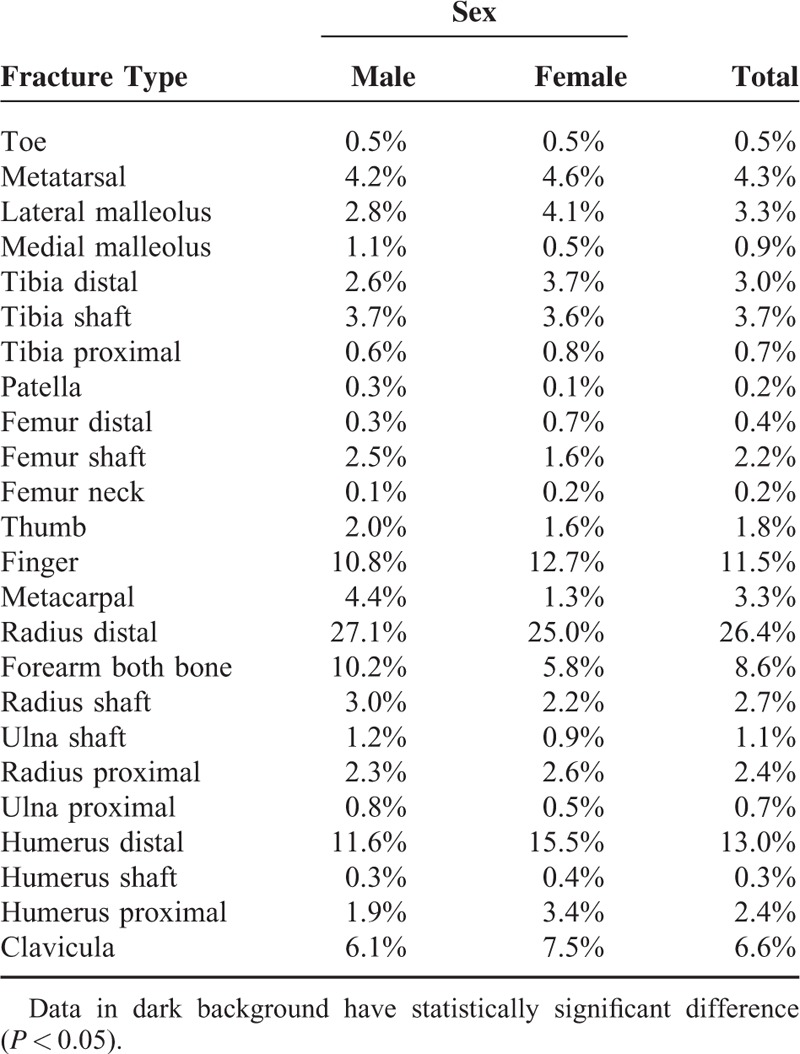
The Ratios of Fractures Within Sexes

**TABLE 2:**
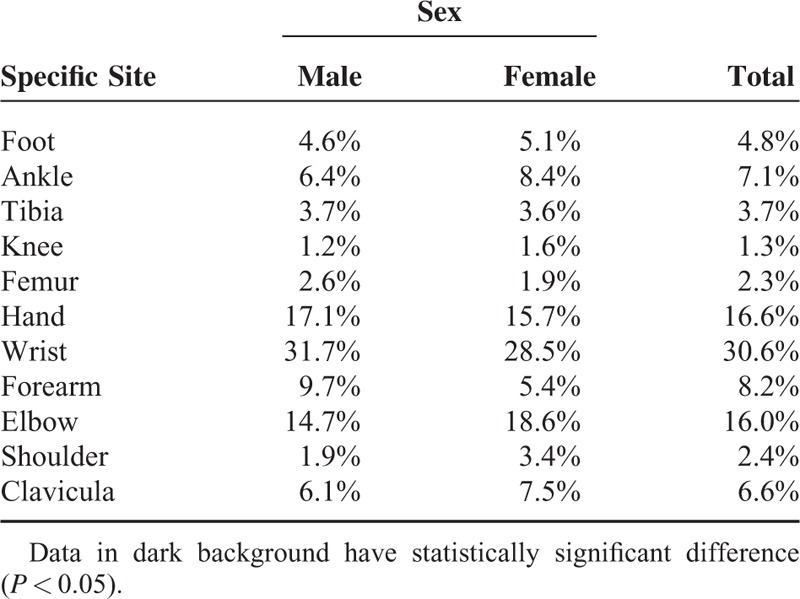
The Ratios of Fracture Sites Within Sexes

### Open Fractures

There were only 22 (0.45%) open fracture cases.

## DISCUSSION

The occurrence of fractures in pediatric ages differs depending on the age, sex, season, and the hour of the day. Types of the fractures show obvious patterns depending on the age regardless of the sex.

Socioeconomic status and risk of pediatric fractures were studied and various relations were found in current studies.^4^ Type of health insurance may give some information about socioeconomic status. In Turkey, “Yeşil Kart” is given to the people with low income. In our study, “Yeşil Kart” holders consist of 1.4% of all the patients. Therefore, our sample does not represent people with lower socioeconomic status.

It has been reported that fracture rates increase as the child gets older.^11^ In addition, gender has an impact on fracture rates in children. Fracture rates decrease after puberty especially in the girls.^3,7,11^ In our study, fractures in the boys made a peak approximately at the age of 13 and then gradually decreased. In the girls, this peak was at the age of 11 (Fig. 1). This might be a consequence of earlier puberty in the girls. A similar graph could be seen in the Cooker study (3).

Girls and boys showed different age-fracture patterns in our study. Although the boys had more fractures gradually till the age of 13, the girls showed a bimodal pattern. From the age of 4 till 7, fracture rates were declining and then increasing till the puberty. Approximately, at the age of 4, gender roles might be affecting the activities of the child and thus girls might not be involved in riskier situations. The increase after the age of 7 might be explained with school engagement. Generally, elbow, shoulder, and ankle site fractures were relatively more in girls, whereas wrist and forearm site fractures were more in boys (Table 2).

Pediatric fractures usually occur at the most active hours of the children. In Sweden, it makes a peak around 15:00.^12^ In India, there is not such a peak but it is rather stable during the day because of the tropical climate.^2^ In our study, fractures occurred more during the hot seasons and less in the cold seasons (Figs. 2 and 3). In addition, they made a peak in the evening around 20:00 and 21:00 (Fig. 4). During the summer, that peak was approximately an hour later than the winter but it showed a similar pattern. This pattern, however, was not visualized in younger children probably because of their activities were not season dependent (Fig. 3). This relatively late-hour peak might be a consequence of the delay between the fracture and the admission. It is common that children did not tell that they had a trauma in the first place to their caregivers and parents might notice their localized pain after they spent some time with their children in the evening.

The notch at the month of June and July could be easily spotted in the graphs (Fig. 3). This sudden decrease at the beginning of the summer is probably because of families going to elsewhere for the summer holiday. Similar findings were found in the countries that have similar holiday customs.^5,7,11^

There was not a strong relation between the fractures and the day of the week. Still the fractures happened less in the middle of the week and more at the beginning and at the end of the week. On Mondays, it was 15.2% and on Fridays and Saturdays, it was 14.9%. Sundays seemed to be calm days also for the children because the least ratio was found on Sundays with a 13.2% (Fig. 4).

Landin et al^7^ classified the fracture-age patterns as the late peak pattern, the bimodal pattern, the decreasing pattern, the rising pattern, the early peak pattern, and the irregular pattern. In our study, some of those patterns were obvious. Wrist and hand fractures showed the late peak pattern, clavicular and elbow fractures showed the early peak pattern, and tibia fractures had an irregular pattern (Fig. 5). These findings were similar to the current studies.^7,11^

In our study, open fractures were less than similar studies with the ratio of 0.45%. Children who have high-energy traumas such as traffic accidents usually have concomitant injuries, which may require comprehensive care that our hospital lacks and thus referrals to our emergency service with that kind of injuries were low. We believed this might be the reason why open fractures were less than similar studies.

Using collaborative data from all medical centers in a well-defined geographical region with known demographics would allow much detailed incidence and risk-assessment studies, which is needed. In this study, these necessary conditions were not met. Thus, the use of the results of this study is limited. These, however, are the most comprehensive data collected in Turkey up to date. Awareness of the age–sex specific patterns would enable more effective planning of providing preventive measures for pediatric injuries. These representative data can be used in several ways, such as to make optimizations in fracture care units of emergency services, scheduling shifts, etc. Also may serve as a calculation aid in relevant studies, which require projections or estimations of fracture properties mentioned above.
